# Preventive measures focused on the urban-rural interface protect rural food-producing communities from SARS-CoV-2

**DOI:** 10.7705/biomedica.6313

**Published:** 2022-10-31

**Authors:** Gina Polo, Diego Soler-Tovar, Luis Carlos Villamil-Jiménez, Carlos Mera

**Affiliations:** 1 Grupo de Investigación en Epidemiología y Salud Pública, Facultad de Ciencias Agropecuarias, Universidad de La Salle, Bogotá, D.C., Colombia Universidad de la Salle Grupo de Investigación en Epidemiología y Salud Pública Facultad de Ciencias Agropecuarias Universidad de La Salle Bogotá D.C Colombia; 2 Center for Natural and Human Sciences, Universidade Federal do ABC, Santo Andre, SP, Brazil Universidade Federal do ABC Center for Natural and Human Sciences Universidade Federal do ABC Santo Andre SP Brazil

**Keywords:** coronavirus infections/prevention and control, communicable disease control, rural population, Colombia, infección y prevención del coronavirus, control de enfermedades transmisibles, población rural, Colombia

## Abstract

**Introduction:**

Rural food-producing communities are fundamental for the development of economic activities associated with sustainability and food security. However, despite the importance of rurality in Colombia, preventive strategies continue to be implemented homogeneously, without considering the dynamics of SARS-CoV-2 in rural food-producing communities.

**Objective:**

To model real areas in Colombia involving rural and urban populations that have intrinsic SARS-CoV-2 transmission dynamics. Characterize rural-urban interactions by means of a parameter that provides different scenarios and allows us to identify interactions capable of preventing SARS-CoV-2 transmission in rural food-producing communities.

**Materials and methods:**

The dynamics of SARS-CoV-2 infection was modeled in five case studies (Boyacá, Caquetá, Cundinamarca, Santander and Sucre) considering urban and rural areas and their interaction (connectivity) in the urban-rural interface. For this purpose, an epidemiological compartmental model considering a classification of individuals according to their economic activity and their epidemiological status was assessed.

**Results:**

Preventive measures focused on the urban-rural interface impact the number of deaths in rural areas. Hence, it is possible to assume that the dynamics of the disease in rural areas depend on the constant interaction with infected individuals from urban areas, which occurs due to the food production dynamics in the urban-rural interface.

**Conclusions:**

Preventive measures should focus on places of high transmissibility and risk for rural communities, such as the urban-rural interface. This work highlights the importance of national heterogeneous preventive measures and the protection of rural communities from the social and economic impacts of SARS-CoV-2.

## Resumen

**Introducción.:**

Las comunidades rurales productoras de alimentos son fundamentales para el desarrollo de actividades económicas asociadas a la sostenibilidad y la seguridad alimentaria. Sin embargo, a pesar de la importancia de la ruralidad en Colombia, las estrategias de prevención continúan siendo implementadas homogéneamente, sin considerar la dinámica del SARS-CoV-2 en estas comunidades.

**Objetivo.:**

Modelar la dinámica del SARS-CoV-2 en poblaciones rurales colombianas. Se quiso caracterizar la interacción rural-urbana mediante un parámetro que proporciona diferentes contextos y permite identificar una interacción rural-urbana capaz de prevenir la transmisión del SARS-CoV-2 en comunidades rurales productoras de alimentos.

**Materiales y métodos.:**

La dinámica de transmisión del SARS-CoV-2 se modeló en cinco estudios de caso (Boyacá, Caquetá, Cundinamarca, Santander y Sucre) considerando áreas urbanas y rurales, así como su interacción (conectividad) en la interfaz urbano-rural. Para ello, se empleó un modelo epidemiológico compartimental que considera una clasificación de los individuos según su actividad económica y su estado epidemiológico.

**Resultados.:**

Las medidas preventivas enfocadas en la interfaz urbano-rural impactan el número de muertes en áreas rurales. Por lo tanto, es posible asumir que la dinámica de la enfermedad en las áreas rurales depende del contacto constante con los individuos infectados de las áreas urbanas, lo que ocurre debido a la dinámica de los sistemas de producción de alimentos en la interfaz urbano-rural.

**Conclusiones.:**

Las medidas de prevención deben enfocarse en lugares con gran transmisibilidad y riesgo para las comunidades rurales, como la interfaz urbano-rural. En este trabajo se destaca la importancia de las medidas preventivas heterogéneas y la protección de las comunidades rurales contra los impactos sociales y económicos del SARS-CoV-2.

In 2020, the World Health Organization (WHO) declared the severe acute respiratory syndrome coronavirus type 2 (SARS-CoV-2) outbreak, originally reported on December 31, 2019, in Wuhan of the Hubei province in China, as a public health emergency ^1^. Until today, the preventive measures against SARS-CoV-2 have focused globally in the homogeneous implementation of mitigation measures such as the interruption of non-essential services for prolonged periods, generating social and economic costs ^2^, or suppression measures such as social distancing that includes the restriction of mobility and the establishment of remote work. On March 17, 2020, the Colombian government issued Decree 417 of 2020, declaring the State of Economic, Social and Ecological Emergency throughout the national territory ^3^. However, differences (heterogeneities) between urban and rural areas were not considered and, therefore, the rural population had difficulties adapting to mobility restriction measures and implementing remote work, suffering negative economic consequences as a result ^2^.

Colombia is one of the Latin American countries with the largest rural population ^4^. According to the *Departamento Administrativo Nacional de Estadística* (DANE), the estimate of inhabitants in rural areas in the country is 22.9% of the total estimated population of 48,258,494 ^5^. Although Colombia, like most countries in the world, has undergone an important urbanization process, only 0.3% of the entire Colombian territory corresponds to urban areas ^6^ and 53% of the population is concentrated in rural territories or in the urban-rural interface ^7^. Although it has heterogeneous conditions, and despite the more than 130,000 deaths reported associated to the SARS-CoV-2 pandemic, and more than six million reported cases ^8^. preventive measures continue to be implemented homogeneously, without considering the virus dynamics in rural food-producing communities and in the urban-rural interface.

Based on the impossibility of implementing suppression (i.e., mobility restriction and remote work) or mitigation (i.e., interruption of activities) measures in rural food-producing communities, this work aims to model real areas in Colombia that have an intrinsic SARS-CoV-2 transmission dynamics in these populations. We characterize rural-urban interaction by means of different scenarios that allows us to identify interactions capable of preventing SARS-CoV-2 transmission in rural food-producing communities.

## Materials and methods

### Study area

**Figure 1:**
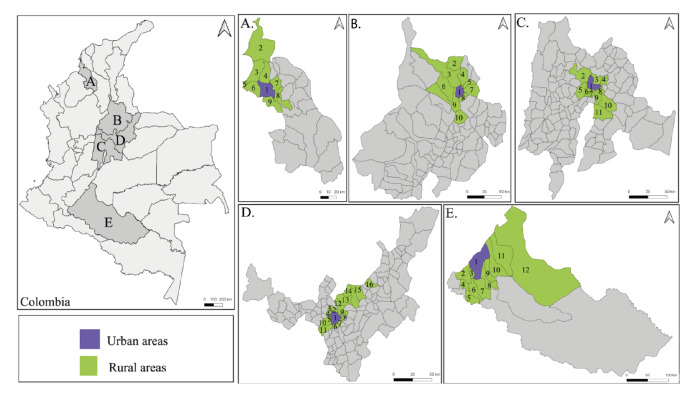
Study area: A) Sucre: 1-Sincelejo, 2-San Onofre, 3-Tolú, 4-Toluviejo, 5-Coveñas, 6-Palmito, 7-Morroa, 8-Corozal, 9-Sampués; B) Santander: 1-Bucaramanga, 2-Playón, 3-Rionegro, 4-Matanza, 5-Charta, 6-Lebrija, 7-Tona, 8-Floridablanca, 9- Girón, 10-Santos; C) Cundinamarca: 1-Zipaquirá, 2-Pacho, 3-Cogua, 4-Nemocón, 5-Subachoque, 6-Tabio, 7-Cajicá, 8-Tocancipá, 9-Sopó, 10-Guasca, 11-Calera; D) Boyacá: 1-Tunja, 2-Motavita, 3-Chiquiza, 4-Sora, 5-Cucaita, 6-Boyacá, 7-Soracá, 8-Chivatá, 9-Oicatá, 10-Samacá, 11-Ventaquemada, 12-Cómbita, 13-Sotaquirá, 14-Paipa, 15-Duitama, 16-Belén; E) Caquetá: 1-Florencia, 2-Belén, 3-Morelia, 4-Albania, 5-Solita, 6-Valparaíso, 7-Milán, 8-Montañita, 9-Paujil, 10-Doncello, 11-Puerto Rico, 12- San Vicente del Caguán.

Colombia has a population of approximately 50 million people, distributed in 32 administrative units called departments, with Bogotá, D.C., as the capital district. In this work, five departments were considered: Boyacá, Caquetá and Cundinamarca (dairy producer), Santander (poultry producer) and Sucre (fish producer) (table 1). In each department, the main urban center was considered, as well as the main neighboring rural municipalities associated with each production system (figure 1).

**Table 1:** Departments and municipalities considered as case studies.

Departament	Municipality	Population	Cases	Deaths	Departament	Municipality	Population	Cases	Deaths
Boyacá	Tunja	179,263	8,473	110 (1.3%)	Cundinamarca	Zipaquirá	146,352	5,268	126 (2.4%)
	Belén	7,532	63	2 (3.2%)		Cajicá	92,967	3,632	43 (1.2%)
	Boyacá	5,118	26	0 (0.0%)		Cogua	24,434	680	31 (4.6%)
	Chiquiza	5,484	34	3 (8.8%)		Guasca	16,934	113	2 (1.8%)
	Chivatá	2,834	13	0 (0.0%)		La Calera	32,917	594	12 (2.0%)
	Cómbita	13,280	465	5 (1.1%)		Nemocón	14,532	208	3 (1.4%)
	Cucaita	3,787	32	1 (3.1%)		Pacho	25,803	296	9 (3.0%)
	Duitama	126,670	4,769	89 (1.9%)		Sopó	28,999	630	16 (2.5%)
	Motavita	5,703	65	4 (6.1%)		Subachoque	16,743	166	5 (3.0%)
	Oicatá	2,890	28	0 (0.0%)		Tabio	24,206	489	8 (1.6%)
	Paipa	34,679	855	16 (1.9%)		Tocancipá	45,714	1177	18 (1.5%)
	Samacá	18,818	444	12 (2.7%) Santander	Santander	Bucaramanga	607,428	28,680	998 (3.5%)
	Sora	3,077	16	1 (6.3%)		Charta	2,888	6	0 (0.0%)
	Soracá	6,068	49	3 (6.1%)		El Playón	14,038	110	11 (10.0%)
	Sotaquirá	8,305	30	0 (0.0%)		Floridablanca	307,896	10,352	421 (4.1%)
	Ventaquemada	16,093	102	3 (2.9%)		Girón	171,904	5,206	184 (3.5%)
Caquetá	Florencia	173,011	9,481	330 (3.5%)		Lebrija	44,169	363	20 (5.5%)
	Albania	4,396	194	4 (2.1%)		Los Santos	14,787	49	6 (12.2%)
	Belén de Andaquíes	11,181	200	4 (2.0%)		Matanza	5,035	31	1 (3.2%)
	El Doncello	19,284	368	15 (4.1%)		Rionegro	27,062	243	13 (5.3%)
	El Paujil	18,464	261	10 (3.8%) Sucre	Sucre	Sincelejo	293,951	11,085	414 (3.7%)
	La Montañita	14,692	205	3 (1.5%)		Corozal	70,853	1,109	54 (4.9%)
	Milán	9,952	107	4 (3.7%)		Coveñas	19,516	506	6 (1.1%)
	Morelia	3,747	77	6 (7.8%)		Morroa	15,858	179	2 (1.3%)
	Puerto Rico	26,282	496	15 (3.0%)		Palmito	15,056	77	1 (5.8%)
	San Vicente del Caguán	52,593	999	42 (4.2%)		Sampués	48,819	468	27 (3.7%)
	Solita	3,815	49	1 (2.0%)		San Onofre	51,109	241	9 (1.5%)
	Valparaiso	7,048	75	4 (5.3%)		Tolú	34,117	329	14 (4.2%)

### Data

The data were obtained from the public reports of mortality due to SARS-CoV-2 reported by the *Instituto Nacional de Salud* of Colombia ^8^ from March 16, 2020, to December 31, 2020 ^8^. As by date, a total of 42,909 deaths were reported, with Boyacá with 28,268 cases (232.20 per 100,000 inhabitants) and 609 deaths (lethality: 2.15%); 14,936 cases (371.68 per 100,000 inhabitants) and 529 deaths (lethality: 3.54%) in Caqueta; 66,254 cases (226.9 per 100,000 inhabitants) and 1,684 deaths (lethality: 2.54%) in Cundinamarca; 66,566 cases (304.7 per 100,000 inhabitants) and 2,361 deaths (lethality: 3.55%) in Santander; and 17,438 cases (192.8 per 100 thousand inhabitants) and 677 deaths (fatality: 3.88%) in Sucre.

### Model

To understand the dynamics of SARS-CoV-2 infection in selected rural food-producing communities, an epidemiological model was considered, classifying individuals according to their economic activity (rural - R: development of activities exclusively in the rural area), urban - U: development of activities exclusively in the urban area) or urban-rural - UR: development of activities in the urban-rural interface) and their epidemiological status (susceptible - S: at risk of developing the disease); exposed - E: infected but not infectious); infectious - I: capable of transmitting the disease; recovered -R: recovered from the disease; or deceased - F: death due to the disease ^9^.

**Figure 2:**
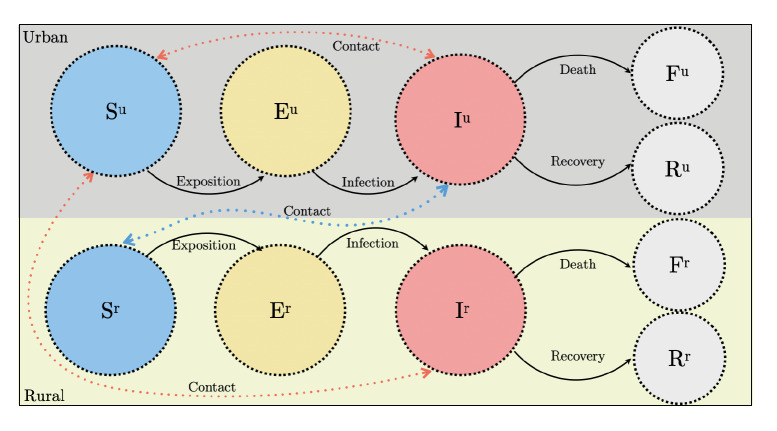
Epidemiological SEIRF model considered for the different case studies. The states correspond to the circles and the transitions between the states are represented by arrows

According to this classification, susceptible individuals residing in rural areas could be exposed to SARS-CoV-2 infection by developing activities at the urban-rural interface through contact with infected individuals residing in urban areas. Likewise, these individuals residing in urban areas could be exposed to the infection through contact with other infected individuals who also reside in urban areas or when carrying out activities in the urban-rural interface. Due to the low contact rate reported in rural areas ^2^, the model does not consider transmission of SARS-CoV-2 among people residing in rural areas. As shown in table 2, this model can be described by twelve different transitions (reactions) from the ten different states and transitions (figure 2).

**Table 2:** Events, reactions, and parameters of the SEIRF epidemiological model

Event	Reaction	Parameter
Exposure of a susceptible individual living in an urban area by contact with infectious individuals in an urban area.	Su → Eu	ϵ^1^
Exposure of a susceptible individual living in an urban area by contact with infectious individuals in the rural area at the urban-rural interface.	Su → Eu	ϵ ^2^
Exposure of a susceptible individual residing in a rural area by contact with infectious individuals in the urban area at the urban-rural interface.	Sr → Er	ϵ ^3^
Infection of an exposed individual living in an urban area.	Eu → Iu	β^1^
Infection of an exposed individual residing in a rural area.	Er → Ir	β ^2^
Recovery of an infectious individual living in an urban area.	Iu → Ru	Y^1^
Recovery of infectious individual residing in rural area.	Ir → Rr	Y^2^
Death of an infectious individual living in an urban area.	Iu → Fu	ð^1^
Death of an infectious individual residing in a rural area.	Ir → Fr	ð ^2^

Su: Urban susceptibles; Sr: Rural susceptibles; Eu: Urban exposed; Er: Rural exposed; lu: Urban infected; Ir: Rural infected; Fu: Urban deaths; Fr: Rural deaths; Ru: Recovered urban; Recovered rural

### Model fitting and scenario simulations

The epidemiological model was fitted to the number of deaths reported by the INS through the adjustment of the model parameters using a maximum likelihood function ^9^^-^^11^. For this adjustment, the number of initial susceptible individuals in each urban or rural municipality was considered according to the population size reported by the DANE ^5^. In this work, we used the maximum likelihood optimizer through the function "mle2" of the package "bbmle" of the computational language R (Bolker, 2020) to estimate this method for the model parameter 0 of each department.

In order to evaluate the impact of preventive measures focused on the urban-rural interface on rural areas, four scenarios associated with variations in the contact rate were considered ^12^^,^^13^ (ϵ = 70%, ϵ = 40%, ϵ = 20%, ϵ = 0%) between the individuals on this interface.

## Results

**Figure 3:**
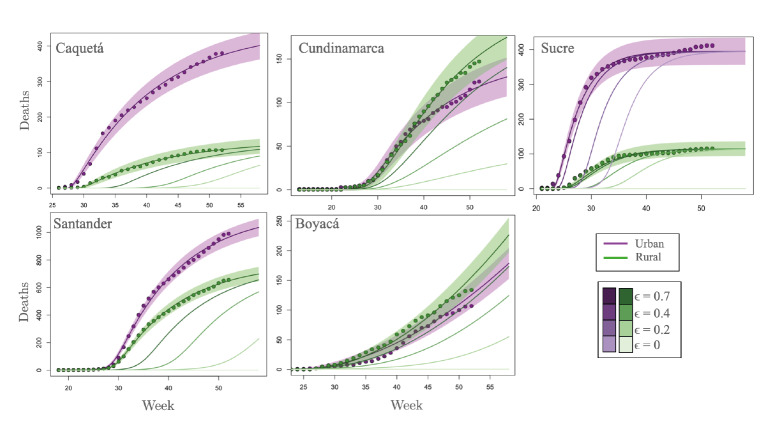
Model fitting to the number of deaths in each of the case study departments considering the selected municipalities of urban and rural areas.

Figure 3 shows the adjustment of the model to accumulated deaths in urban and rural areas reported in each department. This fit demonstrates the ability of the model to reproduce and predict the epidemiological profile of SARS-CoV-2 infection in urban and rural human populations. The list of parameters obtained because of the maximum likelihood function for each department considered in the case studies are found in table supplementary 1 of the supplementary material.

Purple and green dots in figure 3 describe the cumulative number of deaths reported in urban and rural food-producing communities, respectively, while the lines indicate the it of the model to the actual data. There is a clear difference in the epidemiological profile of the pandemic in the different municipalities. Specifically, the irst epidemiological week, defined as the initial week in which the first infections were detected, it is different for each department, as can be observed in the x-axis of figure 3. Within the departments of Sucre and Caquetá, a difference in the first epidemiological week can also be observed for urban and rural populations. However, in Cundinamarca and Boyacá, departments with a high interaction between urban and rural communities, deaths occur simultaneously in rural and urban populations.

There is also a marked difference between the curves describing the accumulated deaths in urban and rural areas in Caquetá, Sucre, and Santander. However, in the departments of Cundinamarca and Boyacá, there is a very similar accumulated number of deaths for rural and urban areas. This difference in the case study departments is mainly because the coefficient that simulates the interaction in the urban-rural interface is markedly higher in Cundinamarca and Boyacá (table supplementary 1).

The importance of the interaction in the urban-rural interface becomes explicit when we consider its variation throughout four scenarios, namely: ϵ = 70%, ϵ = 40%, e = 20%, ϵ = 0%. The modulation of the parameter e counts for the implementation of different preventive measures focused on the urban-rural interface. As observed in figure 3, the preventive measures focused on the urban-rural interface impact the number of deaths in rural areas of all the considered departments, so it can be assumed that the dynamics of the disease in rural areas depend on connectivity (contact rate) at the urban-rural interface. Additionally, as shown in the case of Sucre, preventive measures focused on the urban-rural interface can delay the epidemic peak in urban areas.

## Discussion

Our findings confirm that information about human mobility and connectivity should be a starting point for modeling important dynamic processes in human and animal epidemiology, population ecology, biology, and evolution ^14^^-^^17^. The urban-rural interface is essential for the development of economic activities associated with sustainability and food security ^18^, so it is essential to establish strategic and differentiated preventive measures for these communities. Preventive measures focused on the urban-rural interface may include: mandatory and appropriate use of personal protection items such as face masks ^12^^,^^19^^,^^20^; implementation of transportation systems/ schedules to avoid crowds, including movement restrictions for all family members ^20^^-^^23^; trade products exclusively in open places that social distancing and low contact rates with implementation of sinks and cleaning items that allow constant handwashing ^24^^,^^25^. These recommendations focused on the urban-rural interface will potentially impact the dynamics of the infection, reducing the number of deaths in rural communities.

In the context of a global health emergency, the contributions of epidemiological models are essential to expand the knowledge regarding transmission dynamics, identify patterns and individuals with greater susceptibility, and propose strategic measures that minimize the inevitable adverse health, economic, and social effects ^26^^,^^27^. The findings of the proposed model warn that highly connected areas, such as the urban-rural interface, are vulnerable to infectious outbreaks and can hardly be adapted to the preventive measures proposed worldwide. Measures focused on reducing connectivity or guaranteeing safe interactions in the urban-rural interface can be an effective mitigation strategy to avoid the geographical spread of diseases to rural areas.

Considering the little importance given to the interaction between the rural population, the implementation of preventive measures focused on rural areas is not of fundamental significance in terms of reducing the transmission of SARS-CoV-2. Therefore, people associated exclusively with food production systems do not need to be subjected to the preventive measures implemented by the national government, such as mobility restrictions. Therefore, these areas are exposed to SARS-CoV-2 when trading their products and conducting activities at the urban-rural interface. Thus, in order to reduce exposure to the virus infection, and prevent itstransmission to rural communities in Colombia, measures should focus on places with high transmissibility and risk for rural communities, such as the urban-rural interface.

## Supplementary material

**Table supplementary 1:** List of parameters obtained because of the maximum likelihood function for each department considered in the case studies.

Departament	Parameter	Definition	Unity	Value
Sucre	ϵu	Exposure rate of individuals residing in urban areas.	día-1	1.4e-4
	ϵr	Exposure rate of individuals residing in rural areas.	día-1	1.2e-5
	β	Infection rate.	día-1	0.27
	ρ	Recovery rate.	día-1	0.66
	μ	Death rate	día-1	8.9e-4
Caquetá	ϵu	Exposure rate of individuals residing in urban areas.	día-1	2.1e-21
	ϵr	Exposure rate of individuals residing in rural areas.	día-1	6.2e-4
	β	Infection rate.	día-1	7.6e-2
	ρ	Recovery rate.	día-1	0.99
	μ	Death rate	día-1	2.5e-3
Santander	ϵu	Exposure rate of individuals residing in urban areas.	día-1	2.2e-12
	ϵr	Exposure rate of individuals residing in rural areas.	día-1	5.1e-5
	β	Infection rate.	día-1	7.8e-2
	ρ	Recovery rate.	día-1	0.99
	μ	Death rate	día-1	1.9e-3
Boyacá	ϵu	Exposure rate of individuals residing in urban areas.	día-1	6.8e-3
	ϵr	Exposure rate of individuals residing in rural areas.	día-1	9.3e-3
	β	Infection rate.	día-1	4.5e-4
	ρ	Recovery rate.	día-1	0.79
	μ	Death rate	día-1	6.8e-2
Cundinamarca	ϵu	Exposure rate of individuals residing in urban areas.	día-1	1.3e-4
	ϵr	Exposure rate of individuals residing in rural areas.	día-1	4.2e-5
	β	Infection rate.	día-1	6.1e-2
	ρ	Recovery rate.	día-1	0.98
	μ	Death rate	día-1	1.0e-3
